# Influence of Cross-Linking Degree on Hydrodynamic Behavior and Stimulus-Sensitivity of Derivatives of Branched Polyethyleneimine

**DOI:** 10.3390/polym12051085

**Published:** 2020-05-09

**Authors:** Alina Amirova, Tatyana Kirila, Mikhail Kurlykin, Andrey Tenkovtsev, Alexander Filippov

**Affiliations:** Institute of Macromolecular Compounds of the Russian Academy of Sciences, Bolshoy pr., 31, Saint Petersburg 199004, Russia; tatyana_pyx@mail.ru (T.K.); mike_x@mail.ru (M.K.); avt@hq.macro.ru (A.T.); afil@imc.macro.ru (A.F.)

**Keywords:** polyethyleneimine, cross-linking polymers, molecular hydrodynamics, light scattering, thermoresponsive polymers

## Abstract

Cross-linked derivatives of acylated branched polyethyleneimine containing 2-isopropyl-2-oxazoline units were investigated in chloroform and aqueous solutions using methods of molecular hydrodynamics, static and dynamic light scattering, and turbidity. The studied samples differed by the cross-linker content. The solubility of the polyethyleneimines studied worsened with the increasing mole fraction of the cross-linker. Cross-linked polyethyleneimines were characterized by small dimensions in comparison with linear analogs; the increase in the cross-linker content leads to a growth of intramolecular density. At low temperatures, the aqueous solutions of investigated samples were molecularly dispersed, and the large aggregates were formed due to the dehydration of oxazoline units and the formation of intermolecular hydrogen bonds. For the cross-linked polyethyleneimines, the phase separation temperatures were lower than that for linear and star-shaped poly-2-isopropyl-2-oxazolines. The low critical solution temperature of the solutions of studied polymers decreased with the increasing cross-linker mole fraction. The time of establishment of the constant characteristics of the studied solutions after the jump-like change in temperature reaches 3000 s, which is at least two times longer than for linear polymers.

## 1. Introduction

Thermosensitive polymers have become ever more interesting objects for study because of the wide range of their application in various fields, especially in medicine and biotechnology. For example, they are used as components of drug delivery systems and membranes, diagnostics agents, tissue engineering, etc. [1,2,3,4]. During the recent years, the potential of using thermosensitive polymers of complex architecture in medicine has been demonstrated [5,6]. Of particular interest are stimulus-sensitive systems based on branched polyethyleneimines (PEI).

Polyethyleneimines are widely used in biology and medicine due to the high density of amino groups. Moreover, branched representatives of this chemical class, more compact in comparison with linear analogs, are favored as an effective and affordable nonviral gene delivery vector [7,8,9,10,11], as well as an additive to increase the efficiency of the polymerase chain reaction [12] and DNA degradation protection [13]. Besides, branched PEIs are also used as a matrix, stabilizer, and molecular glue in obtaining metal nanoparticles and metal oxides, semiconductor nanoparticles, and carbon nanotubes [14,15,16,17,18,19,20].

On the other hand, the high content of primary amino groups increases the cytotoxicity of PEI and can cause destabilization of the cell membrane; therefore, chemical modification is used to neutralize the amino groups. In particular, the cytotoxicity of PEI can be reduced by acylation or attaching polymer chains to the amino groups of PEI. This has opened up new ways for branched PEI system applications. In particular, the acetylation, hydroxylation, and carboxylation of PEI reduce polymer cytotoxicity [21]. The substitution of various proportions of the primary amines with alkyl carboxylate moieties having different alkyl chain lengths allowed obtaining nonviral DNA vector [22], and β-cyclodextrin grafting to the macromolecule made it possible to improve nucleic acid delivery [23]. PEI methylation and acylation followed by PEGylation yielded products for gene delivery with improved biocompatibility and lower toxicity, but it reduced transfection efficiency [24], while another acylation route increased transfection as compared to patent PEI [25]. The addition of oligosaccharide chains, including glucose, lactose, and maltose, turned PEI into an effective drug carrier [26].

The properties of the resulting system largely depend on PEI acylation degree. Thus, cross-linked PEIs with low cytotoxicity and high transfection, but varying DNA binding potential depending on the degree of thiolation, were obtained from PEIs with different contents of 2-mercaptopropyl groups [27]. Increased substitution degree reduces the PEI concentration necessary for the high efficiency of the polymerase chain reaction [28,29].

Two methods are used to ensure PEI stimulus sensitivity. The first one involves attaching thermosensitive polymer chains to the PEI molecules. For example, the length of the attached chains of poly(N-isopropyl acrylamide) (P-N-IPAAM), determining conformational rearrangements upon heating the solution, affects the balance between the charged PEI core and the hydrophobic fragments of P-N-IPAAM, which determines the polymer’s ability to bind nucleic acids and cell transfection [30]. Notably, the inclusion of hydrophilic PEI in the macromolecule barely affects the thermosensitivity of the second block; however, due to amino group protonation in the acidic medium, the phase transition is shifted toward higher temperatures as compared to P-N-IPAAM [31]. The block copolymer of PEI and poly-2-ethyl-2-oxazoline formed DNA polyplexes soluble at low pH, which, together with high transfection and low cytotoxicity, makes it promising for nonviral gene therapy [32]. The second approach involves the functionalization of PEI by groups that would provide for the thermosensitivity of the resulting polymer. For example, after sulfopropylation, branched PEI was characterized by an upper critical solution temperature, which depended on the molar mass (MM) of the polymer, the ionic strength of the solution, the acylation degree, and pH [33]. Functionalization with isobutyramide groups, on the contrary, led to the lower critical solution temperature (LCST), which also depended on the acylation degree [34].

In the present work, we studied partially cross-linked derivatives of branched polyethyleneimine, which, along with the monomeric units of ethyleneimine, contain 2-isopropyl-2-oxazoline units ensuring the polymer thermosensitivity of LCST type [35]. The main task was to establish the effect of cross-linking degree on their conformational properties, as well as the processes of self-organization and aggregation of macromolecules in aqueous solutions at varying temperatures, polymer concentrations, and acidity.

## 2. Materials and Methods 

### 2.1. Synthesis of Partially Cross-linked Poly(ethyleneimine)

Branched polyethyleneimine (CAS 9002-98-66, weight-average molar mass *M*_w_ = 25,000 g mol^−1^), hexamethylene diisocyanate, and isobutyroyl chloride (all Sigma Aldrich, St. Louis, MO, USA) were used without further purification. According to NMR spectroscopy data, primary, secondary, and tertiary amines in the branched PEI were present in the ratio of 0.21:0.58:0.21. Using this ratio, the branching degree *DB* ≈ 0.4 was calculated.

^1^H NMR spectra were obtained on a Bruker AVANCE instrument (400 MHz) (Bruker, Billerica, MA, USA) for solutions in deuterated chloroform; chemical shifts were counted relative to the signals of the solvent using Specman ACD/Labs (Advanced Chemistry Development, Inc., Toronto, ON, Canada). For dialysis, CellaSep dialysis bags with MWCO 3500 D (Orange Scientific, Braine-l’Alleud, Belgium) were used.

**Figure 1:**
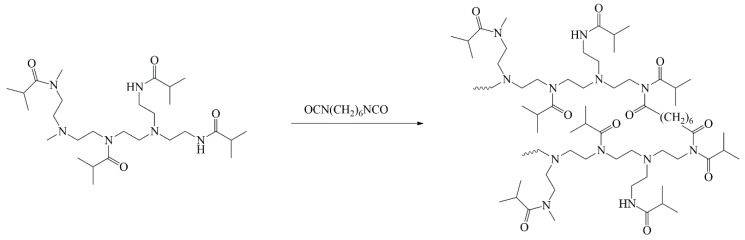
Scheme of synthesis of polyethyleneimines (PEI)-n.

**Figure 2:**
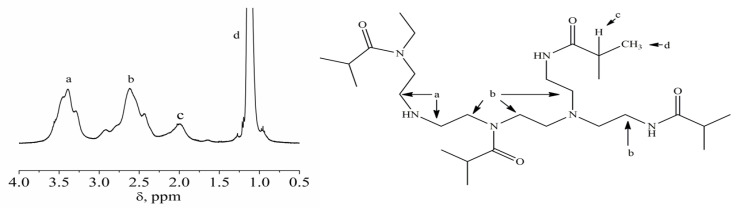
^1^H NMR spectrum and signal assignment for PEI-3.

The acetylated derivative of branched polyethyleneimine (PEI-0), namely, poly-N-isobutyroylethyleneimine, was obtained by acylating polyethyleneimine with isobutyroyl chloride under the conditions of the Einhorn reaction (with methylene chloride solvent and triethylamine acceptor). Cross-linking was performed by adding the calculated amount of hexamethylene diisocyanate as previously described [35]. The synthesis scheme for partially cross-linked polyethyleneimines PEI-n is shown in Figure 1. The obtained samples differed in the cross-linking degree, namely, the molar fraction of the added cross-linker *w*. The *w* values for prepared samples are listed in Table 1. In Figure 2, 1H NMR spectrum and signal assignment for poly-N-isobutyroylethyleneimine partially cross-linked by 1,6-hexamethylene diisocyanate (sample PEI-3 in Table 1) are shown as an example.

### 2.2. Determination of Hydrodynamic Characteristics

**Figure 3:**
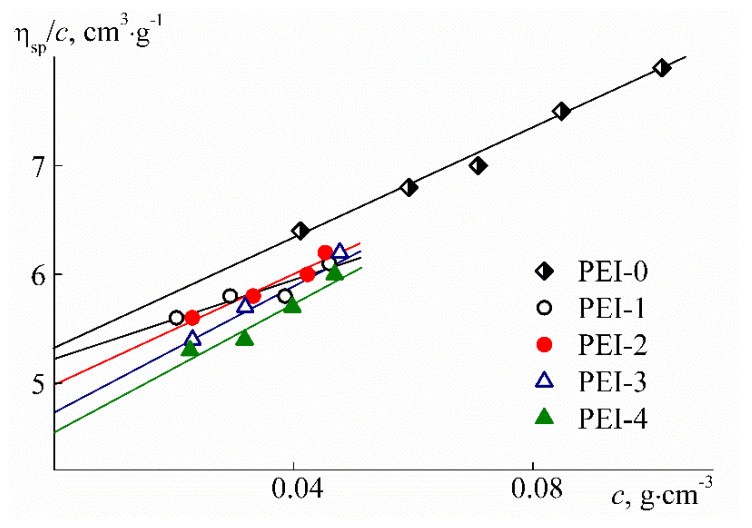
Dependencies of reduced viscosity η_sp_/*c* on concentration *c* for solutions of PEI-0 in dichloromethane and PEI-n in chloroform.

MMs of the synthesized samples were determined previously [35] by the method of static light scattering in dichloromethane (density ρ_0_ = 1.33 g cm^−3^, dynamic viscosity η_0_ = 4.4 × 10^−3^ Poise, and refractive index *n*_0_ = 1.424) for the targeted PEI-0 and chloroform (ρ_0_ = 1.48 g cm^−3^, η_0_ = 5.7 × 10^−3^ Poise, and *n*_0_ = 1.442) for partially cross-linked PEI-n. Before measurements, the solutions and solvents were filtered through the Millipore syringe filter (Merck KGaA, Darmstadt, Germany) with the pore diameter of 0.20 μ. The intrinsic viscosity [η] was measured with the Ostwald-type Cannon–Manning capillary viscometer (Cannon Instrument Company Inc., State College, PA, USA) at 21 °C. To control the solution temperature, a thermostat with the T-100 temperature control unit (Grant, Cambridge UK) was used. The solvent efflux time was 43.4 s for dichloromethane and 48.7 s for chloroform. Dependencies of reduced viscosity η_sp_/*c* on polymer concentration *c* (Figure 3) were analyzed using the Huggins equation

η_sp_/*c* = [η] + *k*′[η]^2^*c* (1)

where *k*′ is the Huggins constant.

**Figure 4:**
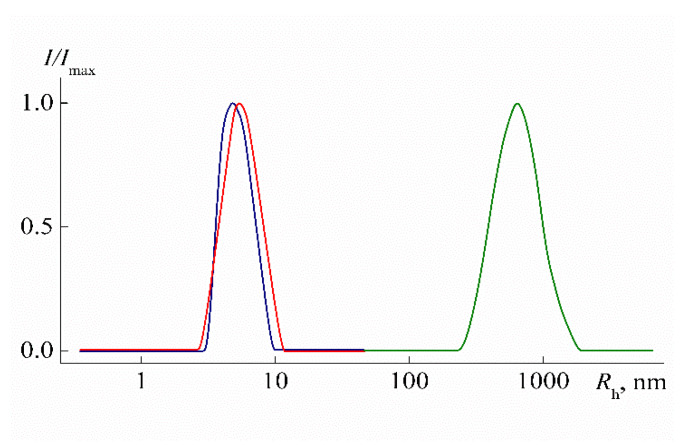
Relative intensity *I*/*I*_max_ of scattered light vs. the size of scattering species *R*_h-D_ for PEI-1 in chloroform (blue) at *c* = 0.0379 g cm^−3^, in water at 10 °C (red) and 19 °C (green) at *c* = 0.0374 g cm^−3^. *I*_max_ is the maximum of light scattering intensity for studied solution.

**Figure 5:**
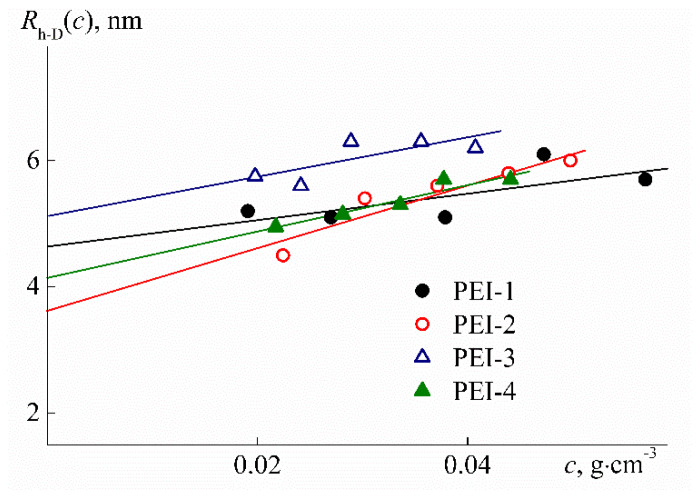
Concentration dependencies of hydrodynamic radius *R*_h-D_(*c*) for solutions of PEI-n in chloroform.

Hydrodynamic radii *R*_h-D_ were determined by dynamic light scattering using the Photocor Complex E instrument (Photocor Instruments Inc., Moscow, Russia) equipped with the Photocor-DL diode laser (wavelength λ = 635.5 nm), Photocor-FC correlator with 288 channels, Photocor-BS device for light backscattering study, and the Photocor-PD detector for measuring the transmitted light intensity. The autocorrelation function was measured with Photocor Software (Photocor Instruments Inc., Moscow, Russia) and processed with DynaLS soft (ver. 8.2.3, SoftScientific, Tirat Carmel, Israel). Solutions of PEI-0 and PEI-n were unimodal over the entire concentration range studied (Figure 4). For all samples, the hydrodynamic radii *R*_h-D_(*c*) of scattering objects that were determined at concentration *c* decreased with dilution (Figure 5). The hydrodynamic radii *R*_h-D_ of macromolecules (Table 1) were obtained by linear extrapolation to zero concentration.

**Figure 6:**
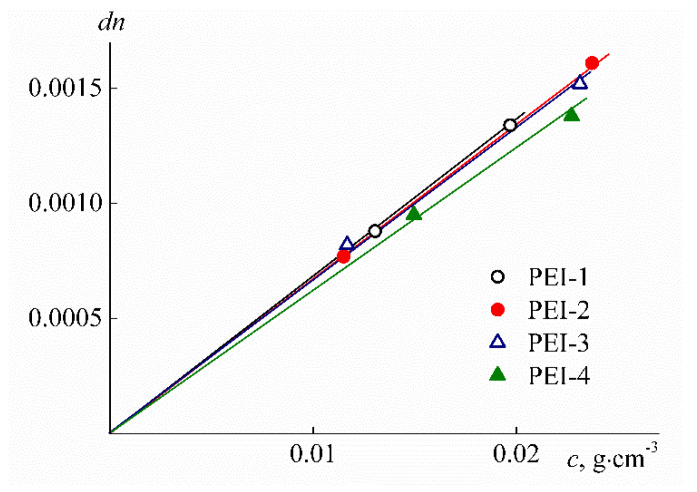
Concentration dependencies of the difference in refractive indices *dn* of the PEI-n solutions and chloroform.

The values for the refractive index increment *dn/dc* were determined based on the concentration dependence slope of *dn* = *n*_s_ – *n*_0_ of the refractive indices *n_s_* for the solution of concentration *c* with *n*_0_ for solvent (Figure 6). The refractive indices *n*_s_ and *n*_0_ were measured with an RA-620 refractometer (KEM, Tokyo, Japan). As seen from Table 1, the values of *dn/dc* are somewhat decreased with the growth of *w.* Thus, increased cross-linker content results in decreasing the refractive index increment, although this change is not significant.

The self-organization of PEI-n molecules in aqueous and water–salt solutions on heating was studied by light scattering, light backscattering, and turbidimetry using the Photocor setup described above. The temperature *T* was varied discretely with a step from 0.5 to 2 °C and maintained with an accuracy of 0.1 °C. The solution concentrations *c* varied in the range of over 10-fold, from 0.0035 to 0.0380 g cm^−3^, and their acidity varied in the range of pH = 2 – 8 at *c* = 0.0280 g cm^−3^. The pH of the initial solution was changed by adding 1 N HCl or 1 N NaOH. The solutions were filtered through hydrophilic PTFE Millipore (Merck KGaA, Darmstadt, Germany) membrane filters with the pore diameter of 0.20 μ.

**Figure 7:**
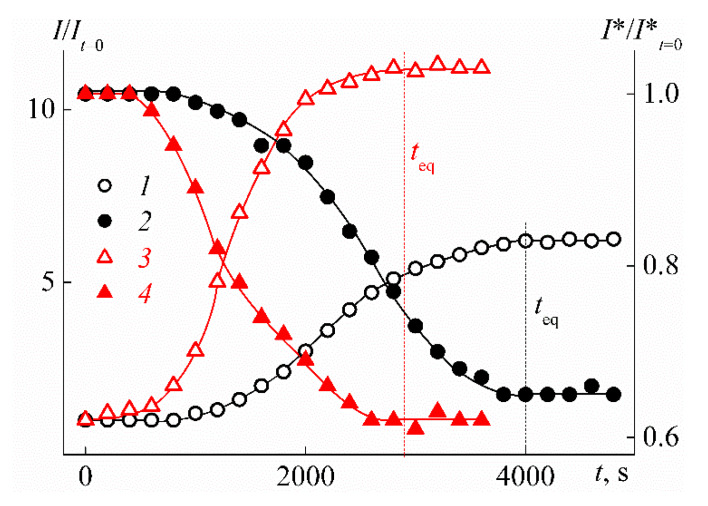
Dependencies of the relative values of light scattering intensity *I*/*I*_t=0_ (1, 3) and optical transmission I*/I*_t=0_ (2, 4) on time *t* for solutions with concentration *c* = 0.0280 g cm^−3^ of PEI-1 at *T* = 16 °C (1, 2) and PEI-2 at *T* = 21 °C (3, 4). *I*_t=0_ and I*_t=0_ are light scattering intensity and optical transmission at *t* = 0, respectively.

The measurement procedure was as follows. After the target temperature was established, the changes in the intensity of the scattered light *I* and the optical transmission I* in time were analyzed. In this case, *I* was measured at the scattering angle of 90°. As a criterion for determining whether the solution has reached the ‘equilibrium’ state, the constancy of *I* and I* in time was chosen. The times *t*_eq_ for establishing the constant characteristics of the solutions were determined accordingly (Figure 7). In ‘equilibrium’ conditions, in addition to *I* and I*, the hydrodynamic radii of the *R*_h_ particles present in the solutions were determined. The light scattering measurements were carried out in the range of scattering angles θ from 45° to 135° to confirm the diffusion nature of the modes and determine the extrapolated values of *R*_h_. The relaxation time τ of a correlation function was measured at scattering angles 45°, 90°, and 135°. The values of scattering wave vector *q* were calculated via equation

(2) $$q=\frac{4\pi n_{0}}{\lambda }sin\frac{\theta }{2}.$$

**Figure 8:**
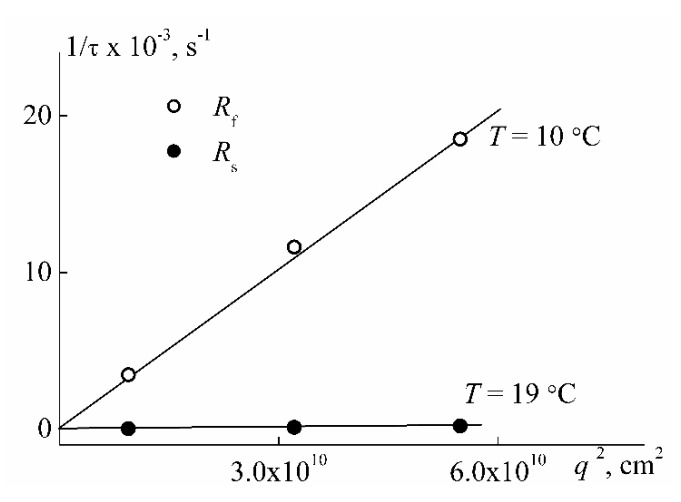
Dependencies of inverse relaxation times on the squared scattering wave vector for PEI-1 in water at 10 °C and 19 °C at *c* = 0.0374 g cm^−3^.

The magnitudes of translational diffusion coefficient *D*_0_ were obtained from dependencies of inverse relaxation time 1/τ on the squared scattering wave vector (Figure 8).

1/τ = *q*^2^*D*_0_ (3)

The Stokes–Einstein equation was used for the calculation of hydrodynamic radius values

(4) $$R_{h}=\frac{kT}{6\pi \eta_{0}D_{0}},$$

where *k* is the Boltzmann constant and *T* is the absolute temperature.

## 3. Results

### 3.1. Structure and Hydrodynamic Behavior of PEI-n in Dilute Chloroform Solution

To determine the conditions for the preparation of poly-isobutyroylethylenimine with a certain degree of acylation, branched PEI was acylated by isobutyroyl chloride within different synthetic methods as well as with the different ratios of reagents. The best results were obtained using the Einhorn reaction [36], which at a given ratio of reagents allowed obtaining a product with a predictable degree of substitution. Diisocyanate was chosen as a cross-linker, keeping in mind the fact that isocyanates react quantitatively with both primary and secondary amino groups at ambient temperature and are relatively resistant to water at room or lower temperature [37]. Additionally, it should be noted that the complete removal of water from PEI and its acylation products is quite problematic.

The interaction of acylated PEI-0 with hexamethylene diisocyanate leads to the formation of both intramolecular and intermolecular cross-links. At low diisocyanate content in the reaction mixture, intramolecular cross-linking is predominantly observed, which manifests itself in the compaction of a macromolecule without significant MM increase. This is exactly what happens with PEI-n samples (Table 1). In this case, intramolecular cycles are formed in macromolecules, whose number increases with an increase in the cross-linker content. The prevalence of intramolecular cross-linking can be explained by a smaller change in entropy as compared to intermolecular cross-linking. Given the equality of changes in enthalpy for intra- and intermolecular reactions, all of the above leads to a still smaller change in the Gibbs free energy, which determines the direction of the process. With an increase in the content of a bifunctional cross-linker in the initial reaction mixture, the probability of intermolecular interactions increases, leading to the formation of large particles with a subsequent loss of polymer solubility.

An adequate interpretation of the results obtained in the study of stimulus sensitivity is impossible without comprehensive information on the characteristics of individual macromolecules. Accordingly, an important research objective was to establish the hydrodynamic characteristics of PEI-n and to analyze the conformation of their molecules.

As seen from Table 1, the refractive index increments *dn*/*dc* decrease slightly with increasing *w*, although this change is not very significant. A decrease in *dn*/*dc* is yet another confirmation that the cross-linker fraction becomes larger, and cross-linking occurs at the macromolecule level primarily. Indeed, the refractive indices of hexamethylene diisocyanate and branched PEI are equal to 1.453 and 1.529, respectively. Therefore, if the cross-linking is intramolecular, an increase in the cross-linker fraction should lead to a decrease in the refractive index increment, which we observe experimentally. 

As known, the Huggins constant characterizes the polymer–solvent hydrodynamic interaction and the hydrodynamic behavior of solutions [38,39,40]. For the PEI-0 and PEI-n under question, *k*′ values lie in the range from 0.7 to 1.5 without changing systematically with the variation of *w*. These values are higher than the usual Higgins constant for linear polymers in good solvents. Elevated *k*′ values are often obtained for polymers with complex architecture, for example, for hyperbranched and star-shaped polymers, as well as for molecular brushes in the region of low MM [41,42,43,44,45]. In particular, for a branched PEI, *k*′ = 0.7 – 0.8 [46]. It can be assumed that the described behavior of *k*′ is explained by the compact size and symmetric shape of macromolecules of polymers with complex architecture.

In organic solvents for the studied samples of PEI-0 and PEI-n, low values of intrinsic viscosity [η] were obtained (Table 1). It is typical for polymers with elevated intramolecular density, such as dendrimers, hyperbranched polymers, polymer stars, and low molar mass polymer brushes with high density of grafting of side chains [41,43,44,45,47,48,49,50,51,52]. Note that at the corresponding MM, the characteristic viscosities of PEI-0 and PEI-n solutions are close to [η] for branched PEI [46,53]. As the cross-linker mole fraction w increases, the intrinsic viscosity of PEI-n decreases, reflecting an increase in intramolecular density. This change is similar to the decrease in [η] at increasing the branching degree and branching functionality in hyperbranched polymers or the arm number in star-shaped polymers [48,54,55,56]. In the case of the studied polymers, the change in intrinsic viscosity is probably caused by an increase in the number of intramolecular cycles, as well as in hyperbranched polymethylsilsesquioxanes [57]. At passage from PEI-0 to PEI-n, the [η] value decreases by about 20%. Therefore, given that, at first approximation, [η] is inversely proportional to the macromolecule density, we can conclude that the latter characteristic also changes by 20%.

**Figure 9:**
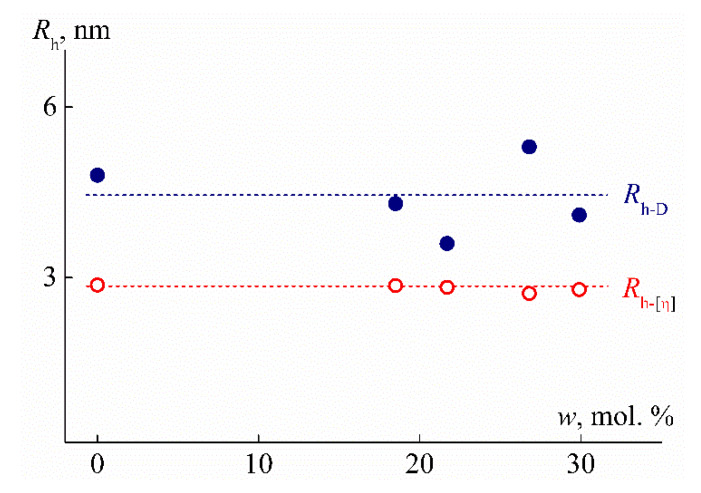
Dependencies of translation hydrodynamic radius *R*_h-D_ and viscosity hydrodynamic radius *R*_h-η_ on cross-linker content *w* for the studied PEI-0 and PEI-n.

To describe the hydrodynamic behavior of hyperbranched macromolecules, the rotation ellipsoid model with slight shape asymmetry is often used [41,47]. The greater the degree of branching of hyperbranched polymers, the better this model describes their hydrodynamic properties. Similarly, the presence of cycles in a branched molecule and an increase in their number brings the shape of the molecule closer to spherical. The volume of a revolution ellipsoid with a closely similar axis length is well proportional to the cube of the average axis length. Therefore, in terms of the model under discussion, it can be expected that the linear dimensions of PEI-n molecules will change by only 6% with an increase in the cross-linker fraction from 0 to 30 mol %. Therefore, it does not seem surprising that the values of the hydrodynamic radius *R*_h-D_ determined by the dynamic light scattering method are independent of *w* (Figure 9). A possible change in *R*_h-D_ lies within the experimental measurement error of this characteristic.

It was found for the so-called viscosity hydrodynamic radius *R*_h-η_, whose values were calculated from the values of the intrinsic viscosity [η] using the Einstein equation, behaves in much the same way (Figure 9):

(5) $$[\eta ]=2.5\bar{v}$$

which can easily yield

*R*_h-η_ = (3*M*[η]/10π*N_A_*)^1/3^, (6)

where $\bar{v}$ is the specific partial volume and *N*_A_ is the Avagadro number. Note that for the studied polymers, the diffusion radius *R*_h-D_ is 1.3–1.9 times larger than the viscous size *R*_h-η_ without a systematic change with increasing *w*. A similar difference is observed quite often both for linear systems [38] and for polymers of complex architecture [41,56,58]. This is because the principle of dimensional equivalence is not fully satisfied during the translational and rotational motion of the macromolecule. Roughly speaking, the molecule ‘flows’ differently in terms of translational diffusion and viscosity. For linear polymers, it results in different values of the Kuhn segment length *A* obtained using the data of viscometry (*A_η_*) and translational friction (*A*_f_). For example, for poly-2-ethyl-2-oxazoline, the difference between *A_η_* and *A*_f_ is 30% [59].

The small values of the radii *R*_h-D_ and *R*_h-η_ confirm the conclusions about the compact size of PEI-n molecules as compared to linear polymers of the same MM. Such values of hydrodynamic radii at the corresponding MM are characteristic of hyperbranched polymers and even dendrimers [41,58,59,60,61,62,63]. The small size and symmetrical shape of PEI-n molecules is also evidenced by the values of the hydrodynamic invariant *A*_0_ (Table 1), calculated by Equation (7) [54,64,65]:

(7) $$A_{0}=\eta_{0}D_{0}{\left(\frac{M\left[\eta \right]}{100}\right)}^{1/3}/T$$

For linear macromolecules, *A*_0_ is constant over a wide MM range. The average experimental values are *A*_0_ = 3.2 × 10^−10^ erg K^−1^ mol^−1/3^ for flexible chain polymers and 3.8 × 10^−10^ erg K^−1^ mol^−1/3^ for rigid chain polymers [38,64] and are in good agreement with the theoretical values of *A*_0_ [38]. On the other hand, for the low molar mass samples of the flexible chain thermosensitive poly-2-ethyl-2-oxazoline, low values of *A*_0_ ~ 2.9 × 10^−10^ erg∙K^−1^∙mol^−1/3^ were obtained [59].

**Figure 10:**
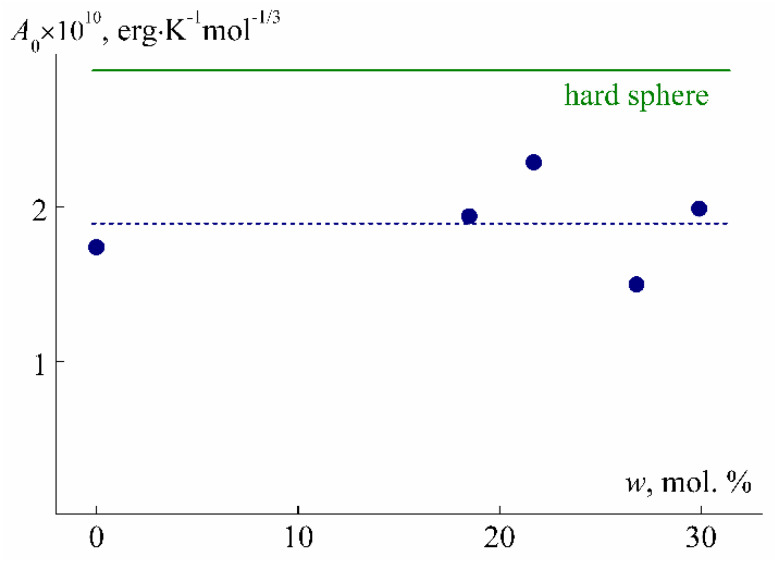
Dependence of hydrodynamic invariant *A*_0_ on cross-linker content *w* for PEI-0 and PEI-n.

In Figure 10, the values of *A*_0_ for the PEI-0 and PEI-n are presented as functions of *w*. The experimental points are rather widely spread, but there is no systematic change in *A*_0_ depending on *w*. The average value *A*_0_ = (1.9 ± 0.2) × 10^−10^ erg K^−1^ mol^−1/3^ is noticeably smaller than the theoretical value for the hard sphere (2.88 × 10^−10^ erg K^−1^ mol^−1/3^). Reduced *A*_0_ was previously found for polymers whose molecules have an increased density and shape approaching spherical, namely, for hyperbranched polymers and dendrimers [41,58,66].

### 3.2. Behavior of PEI-n in Aqueous Solution

The solubility of partially cross-linked branched PEI-n in water depended on the cross-linker content, deteriorating sharply with increasing w. It took about three days to dissolve a sample with *w* = 18.5 mol % with stirring at the temperature of *T* = 10 °C, and a sample with *w* = 21.7 mol % completely dissolved under the same conditions in five days. At *w* ≥ 26.8 mol %, PEI-n did not dissolve within a month at *T* = 6 – 10 °C. Note that a decrease in the solubility of PEI-n was also observed in chloroform, which was manifested in the decrease of the second virial coefficient *A*_2_ from 0.3 × 10^−3^ cm^3^ mol g^−2^ for PEI-1 to −0.2 × 10^−3^ cm^3^ mol g^−2^ for PEI-4. The decreased solubility is due to the increase in the number of intramolecular cycles with increasing *w*, which leads, as mentioned above, to the loss of solubility at *w* > 35 mol %. A similar phenomenon was observed for hyperbranched polymers [57,67,68].

**Figure 11:**
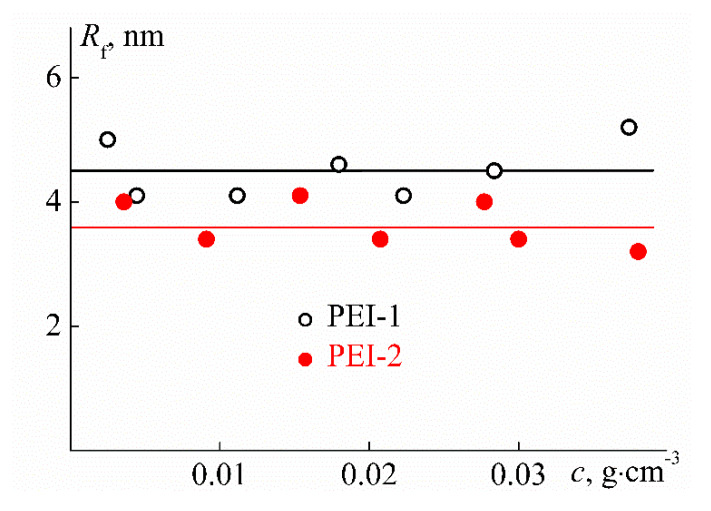
The concentration dependence of hydrodynamic radius *R*_f_ for aqueous solutions of PEI-n samples at 10 °C.

At low temperatures (*T* = 10 °C) in aqueous solutions of PEI-1 and PEI-2, only one particle type was detected by dynamic light scattering, which had the hydrodynamic radius *R*_f_ close to the hydrodynamic size *R*_h-D_ of macromolecules determined in the organic solvent (Figure 9 and Figure 11). Therefore, aqueous solutions of PEI-n were molecularly dispersed, which is typical of stimulus-sensitive polymers that do not contain large hydrophobic fragments [69,70,71,72,73,74]. Moreover, for both samples in aqueous solutions, the radius *R*_f_ with dilution changed only within the experimental error (Figure 11) in contrast to solutions in chloroform. On the other hand, a decrease in *R*_f_ was observed with increasing cross-linker content. The difference in *R*_f_ values for PEI-1 and PEI-2 samples was about 20%.

**Figure 12:**
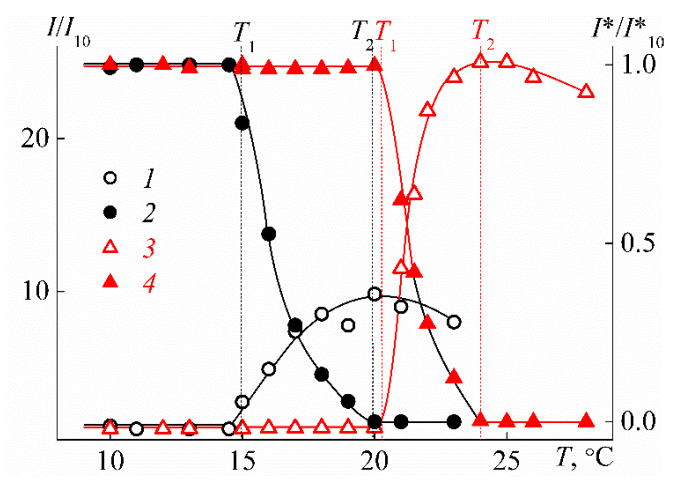
Dependencies of the relative values of light scattering intensity *I*/*I*_10_ (1, 3) and optical transmission I*/I*_10_ (2, 4) on temperature *T* for solutions with concentration *c* = 0.0280 g cm^−3^ of PEI-1 (1, 2) and PEI-2 (3, 4). *I*_10_ and I*_10_ are light scattering intensity and optical transmission at 10 °C, respectively.

**Figure 13:**
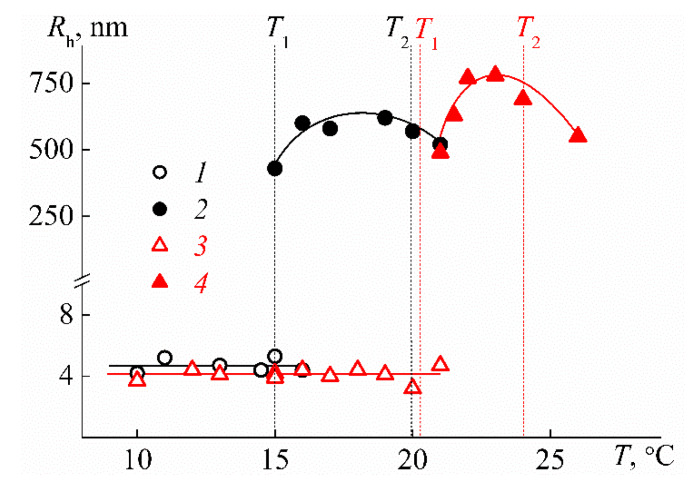
Dependencies of radii *R*_f_ (1, 3) and *R*_s_ (2, 4) on temperature *T* for PEI-1 (1*,* 2) and PEI-2 (3, 4) solutions with concentration *c* = 0.0280 g cm^−3^.

On heating, the phase separation of PEI-n solutions is observed, which manifests itself in a sharp increase in the intensity of scattered light *I* and drop of optical transmittance I* at temperature *T*_1_ (Figure 12). At temperature *T*_2_, the optical transmission falls to zero, i.e., *T*_2_ can be considered as the temperature of phase separation completion determined by turbidimetric data. The solutions of PEI-1 and PEI-2 remained molecularly dispersed up to the temperature of the onset of phase separation *T*_1_ when large aggregates with hydrodynamic radius *R*_s_ were formed in the solution (Figure 13). The phase separation mechanism in PEI-n aqueous solutions involved the dehydration of 2-isopropyl-2-oxazoline units and the formation of intermolecular hydrogen bonds. This led to the formation of aggregates resulting from the association of macromolecules, which, near *T*_1_, cannot be observed by dynamic light scattering. With further heating, the aggregate size first increases, and then, it begins to decrease near *T*_2_, which is probably due to the compaction of PEI-n molecules. Note that a decrease in the molecule size in the vicinity of the phase transition can be observed experimentally for very high-molar-mass samples of thermosensitive polymers [75].

**Figure 14:**
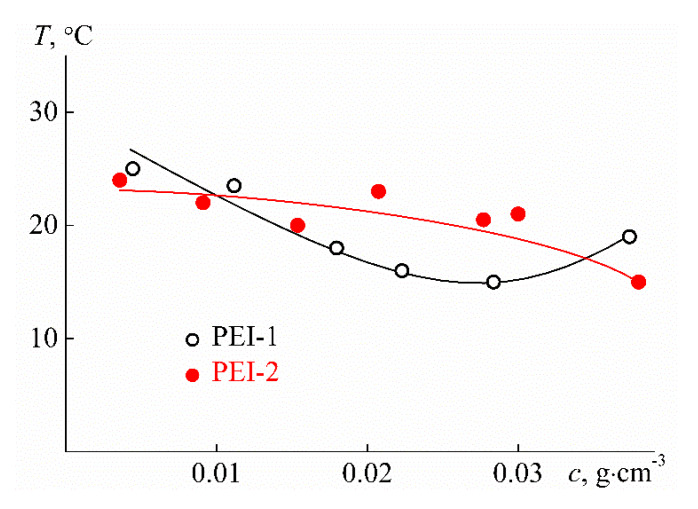
Concentration dependencies of phase separation temperatures *T*_1_ for aqueous solutions of PEI-n.

**Figure 15:**
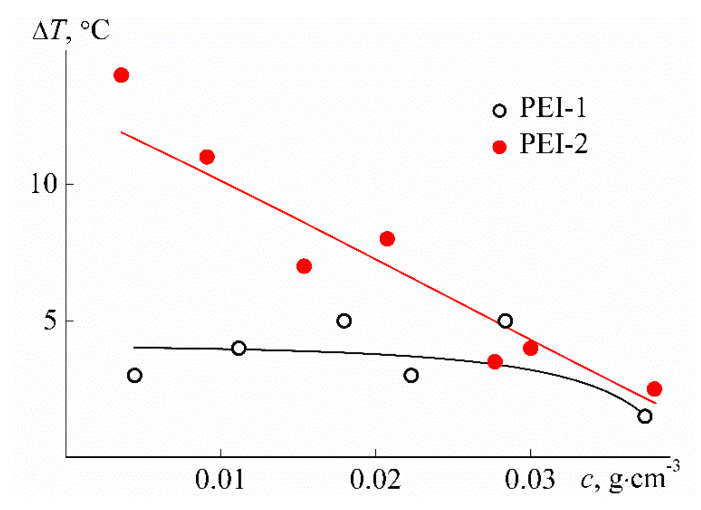
Concentration dependencies of Δ*T* for aqueous solutions of PEI-n.

As can be seen from Figure 14, close phase separation temperatures *T*_1_ were obtained for the samples studied; however, the nature of their change with concentration is different for PEI-1 and PEI-2. For the sample with lower cross-linker content *w*, the *T*_1_ value is minimal at *c* = 0.03 g cm^−3^, indicating that LCST was found close to 15 °C. For PEI-2, the temperature *T*_1_ monotonously decreases with increasing concentration, and in this case, LCST is probably lower than that for PEI-1. Therefore, a rise in the cross-linker content leads to a decrease in LCST. Note that for both samples studied, the phase separation temperatures were noticeably lower than LCST for poly-2-isopropyl-2-oxazoline, which is close to 37 °C for linear and maximum 25 °C for star-shaped polymers [76,77,78,79,80]. A decrease in phase separation temperatures for polymers of complex architecture in comparison with linear analogs is observed quite often [3], but there is no reliable explanation for this behavior yet. The effect of the cross-linker content is also observed when analyzing the width of the phase separation interval, namely, the values Δ*T* = *T*_2_ − *T*_1_. The Δ*T* difference varies slightly with dilution for PEI-1, while a strong dependence of Δ*T* on *c* was found for PEI-2 (Figure 15). In the region of low concentrations, the width of the phase separation interval for PEI-2 is accordingly 10 °C higher than Δ*T* for PEI-1.

As is known, polyethylene imines are polybases. However, no effect of medium acidity on the studied samples was found. In the pH range from 2.0 to 8.1 at low temperatures (*T* < 10 °C), PEI-1 solutions are molecularly dispersed, and the hydrodynamic radius *R*_f_ is independent of pH within the experimental error. In the same way, no systematic change in the phase separation temperatures was observed with varying pH, although the spread in the *T*_1_ values was quite significant, reaching 2.5 °C. Apparently, the number of the remaining unmodified amino groups is too small to lead to conformational rearrangements with varying medium acidity and significantly affects the self-organization of PEI-n molecules.

**Figure 16:**
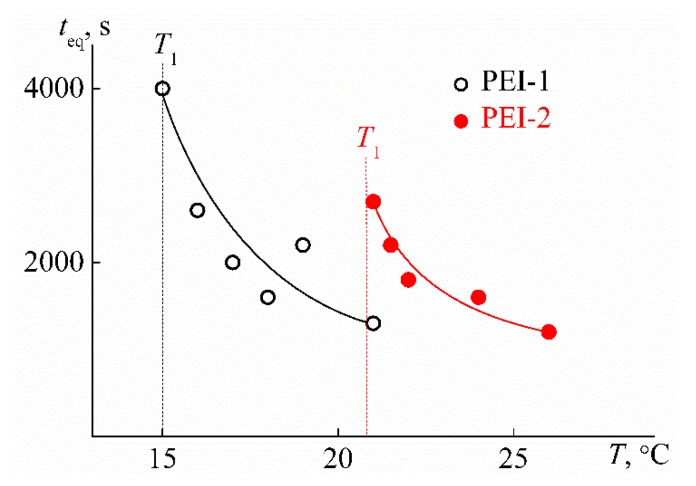
Dependencies of time *t*_eq_ on temperature *T* for solutions of PEI-1 and PEI-2 at *c* = 0.0280 g cm^−3^.

An important feature of the stimulus-sensitive material is the changing rate of its characteristics after exposure. In the case of thermosensitive polymer solutions, this feature is reflected in the time *t*_eq_ that is necessary for the characteristics of the solution to reach constant values after a jump-like change in temperature (Figure 7). For the studied polymers, *t*_eq_ have the maximum value *t*_max_ near the temperature of the phase separation onset *T*_1_, followed by *t*_eq_ decrease on heating (Figure 16). Therefore, it can be assumed that the time *t*_eq_ for PEI-n solutions is determined by the duration of the aggregate formation, which dominates near temperature *T*_1_. Note that a similar pattern in the behavior of *t*_eq_ at *T* > *T*_1_ was previously observed by us for star-shaped poly-2-alkyl-2-oxazolines [81].

**Figure 17:**
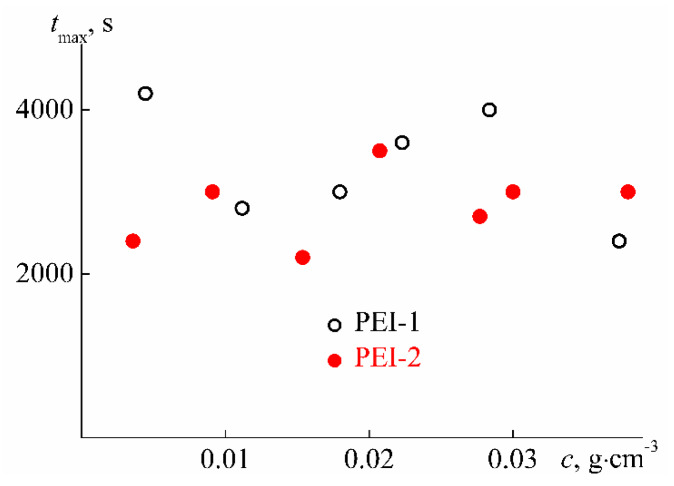
Concentration dependencies of *t*_max_ for aqueous solutions of PEI-n.

In Figure 17, the *t*_max_ values are plotted versus concentration. It can be seen that the maximum value of the time required to establish the ‘equilibrium’ state of the system for both samples does not change upon dilution. The average values *t*_max_ = (2800 ± 300) s for PEI-1 and *t*_max_ = (3300 ± 300) s for PEI-2 coincide within the error range, i.e., the duration of the processes does not depend on the fraction of the cross-linker *w*. As for the absolute values of *t*_max_, they are significantly, sometimes by an order of magnitude, lower than the corresponding characteristic for star-shaped poly-2-alkyl-2-oxazolines and grafted copolymers with side chains of poly-2-alkyl-oxazolines [81,82]. For linear thermosensitive polymers, in most cases, the time *t*_eq_ does not exceed 2000 s [70,83,84,85,86]. The increase in *t*_eq_ during the passage from linear polymers to polymers with complex architecture can be explained by a growth of intramolecular density [81]. For example, the increased density of the hydrophilic outer layer of star-shaped macromolecules prevents the hydrophobic cores from interacting with each other, slowing down the aggregation process. In branched polymers, hydrophobic fragments are more evenly distributed over the macromolecule volume, which may be the reason for the decrease in *t*_eq_ for the studied PEI-n as compared to star polymers.

## 4. Conclusions

The solution properties of partially cross-linked branched PEI-n in chloroform and water were investigated. In both solvents, the solubility worsening of the studied samples was detected with an increase in the cross-linker content, which is caused by an increase in the number of intramolecular cycles. The hydrodynamic characteristics of PEI-n clearly mirror their elevated intramolecular density in organic solvent. It was shown that the cross-linked PEI-n are more compact and symmetric in shape than linear PEI at similar molar masses. The obtained small values of hydrodynamic radius of macromolecules, intrinsic viscosity, and hydrodynamic invariant are characteristic of hyperbranched polymers. The increase in the cross-linker mole fraction leads to a decrease in magnitudes of the intrinsic viscosity of the PEI-n solutions in chloroform that reflects the growth of intramolecular density.

On heating, the aqueous solutions of PEI-n were molecularly dispersed up to the temperature of the phase separation, and the hydrodynamic radii of macromolecules in water and chloroform coincided essentially. At the phase separation temperature, the large aggregates are formed due to dehydration of 2-isopropyl-2-oxazoline units in the PEI-n molecules and the formation of intermolecular hydrogen bonds. The LCST of the PEI-n solutions decreases with increasing cross-linker mole fraction. Moreover, for the samples studied, the phase separation temperatures are noticeably lower than LCST for the linear and star-shaped poly-2-isopropyl-2-oxazolines. Due to the small number of amino groups in the PEI-n molecules, the influence of medium acidity on the characteristics of aqueous solutions was not found. Unexpectedly, with the same MM, the time to establish the equilibrium characteristics of the solution after a temperature change for cross-linked branched PEI-n is less than that for star-shaped poly-2-alkyl-2-oxazolines, despite that the intramolecular density of polymer stars is lower than for cross-linked and hyperbranched polymers.

## Figures and Tables

**Table 1:** Molar masses, structure, and hydrodynamic characteristics of PEI-n.

Sample	*w*, mol %	*M*_w_, G mol^−1^	[η], cm^3^ g^−1^	*dn*/*dc*, cm^3^ g^−1^	*R*_h-D_, nm	*R*_h-η_, nm	*A*_0_ × 10^−10^, Erg K^−1^mol^−1/3^
PEI-0	0	28,000	5.3	0.0964	4.8	2.9	1.7
PEI-1	18.5	28,000	5.2	0.0679	4.3	2.9	1.9
PEI-2	21.7	28,000	5.1	0.0677	3.6	2.8	2.3
PEI-3	26.8	27,000	4.8	0.0666	5.3	2.7	1.5
PEI-4	29.9	30,000	4.5	0.0616	4.1	2.8	2.0
PEI-5	37.9	insoluble					
